# Understanding the discriminant factors that influence the adoption and use of clinical communities of practice: the ECOPIH case

**DOI:** 10.1186/s12913-015-1036-4

**Published:** 2015-09-10

**Authors:** David Lacasta Tintorer, Souhel Flayeh Beneyto, Josep Maria Manresa, Pere Torán-Monserrat, Ana Jiménez-Zarco, Joan Torrent-Sellens, Francesc Saigí-Rubió

**Affiliations:** Centre d′Atenció Primària la Salut, Institut Català de la Salut, Badalona, Spain; Unitat de Suport a la Recerca Metropolitana Nord, IDIAP Jordi Gol, Santa Coloma de Gramenet, Spain; Universitat Autònoma de Barcelona, Bellaterra, Cerdanyola del Vallès, Spain; Departament de Ciències Mèdiques, Universitat de Girona, Girona, Spain; Economics and Business Studies and Internet Interdisciplinary Institute, Universitat Oberta de Catalunya, Barcelona, Spain; Department of Health Sciences and Internet Interdisciplinary Institute, Universitat Oberta de Catalunya, Barcelona, Spain

## Abstract

**Background:**

The aim of the study presented in this article is to analyse the discriminant factors that have an influence on the use of communities of practice by primary and specialist healthcare professionals (physicians and nurses) for information sharing. Obtaining evidence from an ex-ante analysis to determine what factors explain healthcare professionals’ clinical community of practice use allows aspects of its use to be identified.

**Methods:**

A theoretical model based on a modified technology acceptance model was used as the analysis tool, and a discriminant analysis was performed. An ad-hoc questionnaire was designed and sent to a study population of 357 professionals from the Badalona-Sant Adrià de Besòs Primary Care Service in Catalonia, Spain, which includes nine primary care centres and three specialist care centres. The study sample was formed by the 166 healthcare professionals who responded.

**Results:**

The results revealed three main drivers for engagement in a CoP: First, for the whole sample, perceived usefulness for reducing costs associated with clinical practice was the factor with the greatest discriminant power that distinguished between users and non-users, followed by perceived usefulness for improving clinical practice quality, and lastly habitual social media website and application use. Turning to the two sub-samples of healthcare professions (physicians and nurses, respectively), we saw that the usefulness stemming from community of practice use changed. There were differences in the levels of motivation of healthcare professionals with regards to their engagement with CoP. While perceived usefulness for reducing costs associated with clinical practice was the main factor for the physicians, perceived usefulness of the Web 2.0 platform use for communication for improving clinical practice quality and perceived ease of use were the main factors for the nurses.

**Conclusions:**

In the context of communities of practice, the perception of usefulness of Web 2.0 platform use for communication is determined by organisational, technological and social factors. Specifically, the position that professionals have within the healthcare structure and particularly the closer healthcare professionals’ activity is to patients and their professional experience of using social networks and ICTs are crucial to explaining the use of such platforms. Public policies promoting Web 2.0 platform use for communication should therefore go beyond the purely technological dimension and consider other professional and social determinants.

## Background

In the current context of healthcare spending containment, the role of primary care (PC) is fundamental in preventing unnecessary referrals and reducing waiting lists [1–3], and experiencing long term conditions that are often complex with multiple co-morbidities. However, a characteristic feature of PC surgeries is that they have to attend to a high number of patients suffering from many different health problems, whose clinical complexity is considerable [3–6]. This means that healthcare professionals have to deal with several aspects at once, which may raise a multitude of issues in day-to-day clinical practice [7–10] that require an effective system to search for information and solve problems [11, 12].

Clinical sessions and individual conversations (in person and over the phone), together with specialist care, are options that allow such issues to be resolved. Given that the health system is at saturation point, communication between PC and specialist care is not easy, quick or effective, and it leads to many referrals to specialist care (hospitalisation or specialist outpatient consultations) that generally entail excessive delays for appointments [10, 13].

For some time now, several approaches that draw on the advantages that telemedicine offers with regard to improving communication between PC and specialist care have been tried out [14, 15], with significant benefits in terms of efficiency, cost-effectiveness and improved medical care [16]. Other studies that have assessed healthcare professionals’ levels of satisfaction with the use of different telemedicine tools applied to communication have shown a high degree of confidence in terms of improved medical care and use of time [17–19]. Newer still is the creation of communities of practice (CoP) in the field of healthcare. These are described as a group of people who share an interest in a domain of human endeavour and engage in collective learning that creates bonds among them, sharing knowledge and solving problems in the process [20], and giving healthcare professionals working at different levels of care the chance to collaboratively build knowledge [21, 22]. These virtual communities have not only proven capable of solving problems in a much simpler way, but also of improving the functioning of organisations by generating the kind of tacit knowledge that emerges from interactions among colleagues [23].

In order to understand the effects of clinical CoPs use on health outcomes, it is crucial to analyse the prior step, that is to say, to perform an ex-ante analysis to determine what factors explain physicians’ clinical Communities of Practice use. Obtaining and comparing this evidence represents an important contribution to the literature, in the sense that it will allow the determinants of clinical CoPs use to be evaluated.

The aim of this article is to analyse the discriminant factors that have an influence on the use of CoPs by PC and specialist healthcare professionals (physicians and nurses) for information sharing. To that end, it presents the use of the Web 2.0 Platform for Communication between PC and specialist care called ECOPIH, from the acronym in the Catalan language of “Eina de Comunicació Online entre Primària i Hospitalària” (“Online Communication Tool between Primary and Hospital Care” in English). It is a clinical CoP that includes healthcare professionals from PC centres and specialists from several hospitals in Badalona and Sant Adrià de Besòs (two cities in the Barcelona metropolitan area, Spain) and allows online interaction and communication among healthcare professionals from primary and specialist care to seek advice on clinical cases [24].

## Methods

### Hypothesis and model

The technology acceptance model (TAM) is the theoretical proposal most widely applied to research into the acceptance of new information technologies in the professional sphere [25–31]. In particular, the model has the capacity to robustly explain variance in the intention to use information and communication technologies (ICTs) and ICT use behaviour, taking into account the individual’s perceptions of technology. Specifically, in the original model proposed at the end of 1980, these are [1] perceived usefulness and [2] perceived ease of use [32, 33]. According to Davis, perceived usefulness refers to the extent to which a person believes that using a particular system will improve his or her performance at work. Whereas perceived ease of use refers to the extent to which a person believes that using a particular system will render the effort to perform his or her tasks less arduous [25–28]. However, some studies conducted in the field of healthcare have shown that Information and Communication Technologies use has a two-fold usefulness. Firstly, it improves clinical practice quality [34, 35], and secondly, it reduces the economic, time and human costs associated with clinical practice [36, 37]. Thus, we propose the following hypothesis in relation to the influence that perceived usefulness has on use:

*H1. The perceived usefulness of ECOPIH has an influence on the healthcare professional’s intention to use it.*

*H1.1. The perception of improved clinical practice quality has an influence on the healthcare professional’s ECOPIH use.*

*H1.2. The perception of reduced costs associated with clinical practice has an influence on the healthcare professional’s ECOPIH use.*

Regarding the second variable, the TAM shows how the perceived ease of use of ICTs has a two-fold effect on the individual. Firstly, a greater intention to use technology; and secondly, greater perceived usefulness of it. In this respect, Davis et al. [26] argued that improved ease of use could be instrumental in contributing to an increase in medical professionals’ efficiency. In this respect, the second hypothesis proposed is related to the influence of the ease of use of ECOPIH, and it is established in the following terms:

*H2. The perceived ease of use of ECOPIH in clinical practice has an influence on the healthcare professional’s use of it.*

Despite its widespread acceptance, this model has a series of limitations that mainly stem from the fact that it does not take the influence of other types of variables into account. Bagozzi [38] and Venkatesh et al. [39] underscored the need to increase the explanatory power of this model by incorporating additional variables. According to Davis, identifying variables like these in the TAM can increase the explanatory power of the system users’ acceptance [27, 40]. This is particularly important to the development of TAMs in the field of healthcare, where the consideration of variables relating to information security and protection constitutes one of the main incentives for or barriers to technology acceptance [30, 40–42]. In this respect, the third hypothesis is proposed in the following terms:

*H3. The perception of information security and confidentiality offered by ECOPIH use has an influence on the healthcare professional’s use of it.*

Finally, it should be noted that healthcare professionals use ICTs in their professional and personal lives. As ICT users, they may have their preferences when it comes to using different digital devices and applications in their professional and personal lives. In this respect, social aspects [43] play a role in defining the ICT user profile, as do other circumstantial variables such as experience and training [44]. The development of mixed-approach models comprising elements that refer to both the user profile and to technology, such as Parasuraman’s theory of technology readiness (TR) [45], allowed us to consider the need to incorporate elements that refer to the user profile and the user’s relationship with ICTs. That is why we incorporated the healthcare professional’s ICT user profile as an explanatory factor of his or her intention to use Information and Communication Technologies at an advanced level in his or her usual practice. In this respect, we propose the following hypothesis in relation to the influence of the healthcare professional’s profile as an individual:

*H4. The ICT user profile of the healthcare professional’s – as an individual, in his or her personal life – has an influence on ECOPIH use.*

*H4.1. The use of different mobile devices has an influence on ECOPIH use.*

*H4.2. The use of different social media websites and applications has an influence on ECOPIH use.*

**Fig. 1:**
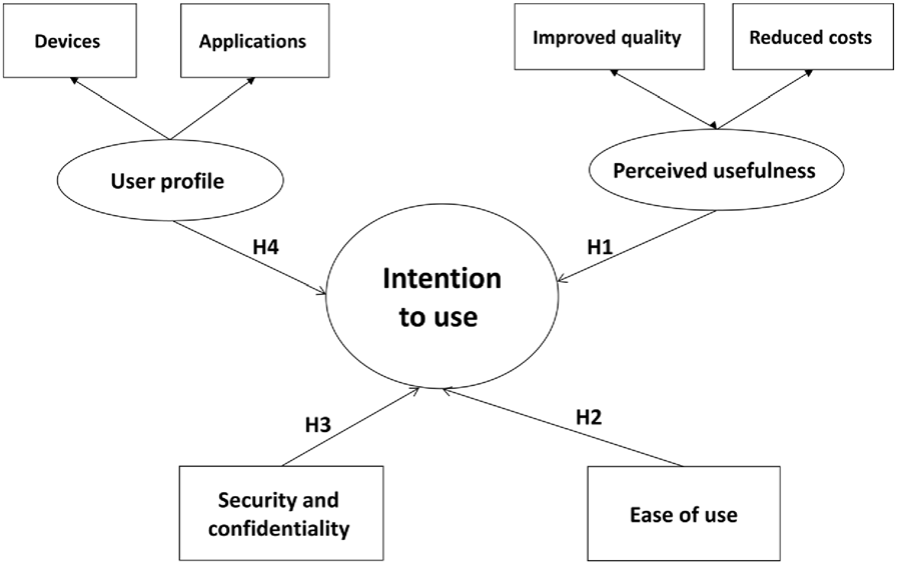
Intention to use ECOPIH TAM

Figure 1 summarises the variables that foster ECOPIH use by the healthcare professionals (Fig. 1).

### Data collection, empirical methodology and validation

As mentioned previously, this study assesses the Web 2.0 Platform for Communication between PC and specialist care (ECOPIH). It is a clinical CoP that uses the e-Catalunya platform (http://ecatalunya.gencat.cat) as its content management system. It enables users to share information quickly and easily due to its numerous applications, incremental deployment options and easy integration into well-known environments. A series of sub-groups have been created on the system (one for each active specialty). The following tools are available within each group: i) a forum where queries about clinical cases are raised for consultation; ii) a document repository and an image repository; iii) a blog where news that people want to share is published; iv) a calendar and a tool for online document editing [44]. The forum rules of use include respect for the confidentiality of the patient and the identification of the healthcare professional involved. Posts are not moderated prior to publication, though they are reviewed to ensure that they comply with the rules. The platform has a notification system that allows members to receive daily, weekly or monthly e-mails, containing updates on news available on ECOPIH [24].

The research presented in this article is the result of a collaboration between the Badalona-Sant Adrià de Besòs Primary Care Service (PCS) in Catalonia, Spain, and the Open University of Catalonia (UOC), Spain. The Badalona-Sant Adrià de Besòs PCS includes nine PC centres and three specialist care centres: Germans Trias i Pujol University Hospital, Badalona Municipal Hospital and the Barcelonès Nord International Health Unit, with a total of 624 healthcare professionals. These centres serve 227,151 inhabitants.

The data collection instrument used was an anonymous survey that included a combination of open and closed questions. The questionnaire was designed collaboratively by both organisations, and it was divided into three sections: 1) sociodemographic and professional background; b) access to and use of ICTs in professional and personal settings; and c) perceptions and use of ECOPIH. Two versions of the questionnaire were produced; one for PC professionals and another for specialist healthcare professionals. The questionnaires were anonymous and optional, and potential respondents were informed about the scientific objectives pursued and the confidentiality of data obtained.

An invitation to complete the questionnaire was sent by e-mail to 357 healthcare professionals bound by common practice in caring for the population of Badalona-Sant Adrià de Besòs PCS and who were regular users of ECOPIH. Among the 357 were 66 supply staff and resident physicians, and 89 specialist professionals could freely send the questionnaire to other colleagues. The questionnaire was completed by a total of 166 healthcare professionals, who formed the study sample and determined the overall response rate, which was 46.4 %, slightly lower than half of the eligible study population. The high response rate reflected by this percentage ensured that the sample was representative of the ECOPIH community population. This was also confirmed after analysing the sociodemographic profile of the individuals comprising the sample (see Table 2).

Likewise, the finite size of the population meant that it was possible to work with low margins of error (+5.6 %, 95 % confidence level), thus allowing the results obtained to be extrapolated.

The fieldwork was carried out between 1 December 2011 and 31 January 2012. Table 1 shows the study specifications. Table 2 shows the variables used in the study. The model described in section 1 presents the professional use of ECOPIH as a dependent variable. The rest of the model’s variables are explanatory and act as independent variables in the model (see Table 2). It should be noted that the two variables used to measure perceived usefulness – *Perceived usefulness for improving clinical practice quality*, and *Perceived usefulness for reducing costs* – were obtained from principal component analysis. The multidimensional nature of these variables suggested that exploratory factor analysis (EFA) should be performed to calculate them. EFA is a data dimensionality reduction technique. Starting with the analysis of a set of original variables, it seeks to determine the smallest number of dimensions capable of explaining the maximum amount of information contained in the data [46].

**Table 1:** Study specifications

Universe	
Universe	357 healthcare professionals
Sample	166
Margin of error	5.6 % (*p* = q) 95 % confidence level
Data collection method	Questionnaire
Sampling method	Random
Fieldwork	December 2011

**Table 2:** Variables used in the study

Model variable		
ECOPIH use		The healthcare professional uses ECOPIH. Dichotomous variable, where 0 = no and 1 = yes.
Perceived usefulness of ECOPIH	Perceived usefulness for improving clinical practice quality (PU1)	Metric variable obtained from a principal component analysis (see Table 3) determining the extent to which the healthcare professionals perceived that ECOPIH use improved clinical practice quality.
	Perceived usefulness for reducing costs (PU2)	Metric variable obtained from a principal component analysis (see Table 3) determining the extent to which the healthcare professionals perceived that ECOPIH use reduced clinical practice costs (in time and effort invested in getting hold of information).
Perceived ease of use of ECOPIH		Variable measured on a 5-point Likert scale indicating the healthcare professionals’ perceived ease of use of ECOPIH.
Security and confidentiality		Variable measured on a 5-point Likert scale indicating the level of patient data security and confidentiality that ECOPIH has.
Healthcare professional profile		Dichotomous variable indicating the individual’s professional profile. 1 = physician and 0 = nurse.
Ict user profile	Mobile device use	Categorical variable indicating the extent to which the ICT user uses different types of mobile device. 1 = low, 2 = medium, 3 = high, 4 = advanced.
	Social media website and application use	Categorical variable indicating the extent to which the ICT user uses social media technologies (access to social networks). 1 = low, 2 = medium, 3 = high, 4 = advanced.
Professional specialization level		Dichotomous variable indicating professional specialisation level in the healthcare sector. 0 = nurse, 1 = physician.

In order to extract the factor dimensions, nine variables were considered in total. Each of them was related to the healthcare professionals’ perceived benefits of using ECOPIH. In particular, some of these variables refer to the benefits relative to quality improvement, while others are relative to the cost reduction that ECOPIH can offer its users. The variable *Perceived usefulness of ECOPIH* has two dimensions: *Perceived usefulness for improving clinical practice quality*, and *Perceived usefulness for reducing costs*. Both dimensions were obtained using principal component factor analysis. By conducting a set of statistical tests, we were able to establish the suitability of the analysis, as well as the reliability of the scale. All the variables of the correlation matrix showed high correlations and the value of their determinant was 0.041. The Kaisser-Meyer-Olkin index value was 0.924 and Bartlett’s test of sphericity value was 1983.717, with a significance of 0.000. This analysis explained 86.846 % of the variance, and Cronbach’s alpha values were higher than 0.81 in all the scales. According to Nunnally [47], this indicator must have values higher than 0.7 in general, and 0.6 in the case of new scales. Thus, it is possible to assume that the scales used were reliable. Additionally, the content and construct scales’ discriminant, convergent and nomological validity were also addressed. With regard to the content, the scales were developed following a major review of the literature (Table 3).

**Table 3:** Factor analysis results. Perceived usefulness of ECOPIH

	Improved quality	Reduced costs
ECOPIH allows the number of referrals to be reduced	0.950	
ECOPIH allows the quality of referrals to be improved	0.967	
ECOPIH allows patient care to be improved	0.958	
ECOPIH improves communication between levels of care	0.921	
The platform that ECOPIH uses (e-Catalunya platform) is satisfactory in terms of displaying information.		0.891
The time it takes to get answers to my queries on ECOPIH is satisfactory for my needs		0.831
The quality of ECOPIH content is good		0.911
Access to specialists to consult on particular cases is easy		0.891
The ability to look up old cases for resolving new issues is useful		0.912
Eigenvalue	3.602	1.938
Variance explained	68.769	18.077
Cronbach’s alpha	0.963	0.932

The results obtained from the data analysis are shown in the following sections. In order to establish the healthcare professional’s profile, univariate analyses were performed on the different sociodemographic and ICT use variables for the selected sample. In addition, in order to identify the discriminant variables of ECOPIH use for the sample as a whole and for the sub-samples (physicians and nurses, respectively), discriminant analysis was performed.

### Ethics

The ECOPIH project has been reviewed and approved by the Ethics Committee and Clinical Research of the Primary Care Research Institute IDIAP Jordi Gol (Barcelona, Spain) [24].

The ECOPIH project follows the national regulation (Spanish Law 14/2007, 3rd July, Biomedical Research) and International regulations for ethical issues (Helsinky and Tokio declarations). The features of the intervention exclude the study to meet the national regulations for clinical trials. On the other hand, confidentiality is guaranteed under the Personal Data Protection Law (15/1999, 13th December).

All Participants were informed about the research objectives before completing the questionnaire. Furthermore, it was also specified that the information obtained would be used for further research purposes. By completing this questionnaire, participants were giving their implied consent to participate in the study.

From ECOPIH platform is not possible to access the medical history of patients.

## Results

### Healthcare professional sociodemographic profile

The sample comprised a total of 166 healthcare professionals. Of these, 65.6 % were physicians, as shown in Table 3. Regarding gender, 68.1 % were female. Based on professional profile (physician *versus* nurse), we found that these percentages differed; just over half of the physicians (56.9 %) were female whereas the large majority of nurses (89.5 %) were female (see Table 4).

**Table 4:** Descriptive statistics of the sample

		Sample	Physicians	Nurses
		166	109	57
Gender	Female	113 (68.1 %)	62 (56.9 %)	51 (89.5 %)
	Male	53 (31.9 %)	47 (43.1 %)	6 (10.5 %)
Age	20–35 years	31 (18.7 %)	24 (22.0 %)	7 (12.3 %)
	35–45 years	42 (25.3 %)	28 (25.7 %)	14 (24.6 %)
	45–55 years	52 (31.3 %)	34 (31.2 %)	18 (31.6 %)
	>55 years	41 (24.7 %)	23 (21.1 %)	18 (31.6 %)
Place of work	Primary Care	140 (84.3 %)	87 (79.8 %)	53 (93.0 %)
	Specialist Care	26 (15,7 %)	22 (20,2 %)	4 (7 %)
Mobile device use	Low	42 (25.3 %)	24 (22.0 %)	18 (31.6 %)
	Medium	62 (37.3 %)	38 (34.9 %)	24 (42.1 %)
	High	51 (30.7 %)	39 (35.8 %)	12 (21.1 %)
	Advanced	11 (6.6 %)	8 (7.3 %)	3 (5.3 %)
Social media website and application use	Low	63 (38.0 %)	42 (38.3 %)	21 (37.5 %)
	Medium	98 (58.9 %)	65 (59.8 %)	32 (57.1 %)
	High	5 (3.1 %)	2 (1.9 %)	4 (5.4 %)

Most professionals had many years of work experience as 56 % of the sample were over 45 years of age. Based on professional profile, these percentages did not significantly differ; 52.3 % of the physicians were in this age segment whereas 63.2 % of the nurses were in this age segment. It should be noted that 31.6 % of the nurses were in the over-55 age segment whereas 22 % of the physicians were in the under-35 age segment. Finally, the vast majority of both physicians (79.8 %) and nurses (93 %) worked in PC.

Overall mobile devise use among the study population ranged from medium to high, with physicians tending to have slightly higher use that nurses, who tended to be medium to high; this segment accounted for 68 % of the sample. The physicians’ use of them also tended to be medium to high, with 70.7 % of the sample concentrated in this segment. In contrast, the nurses’ use of them was medium to low, with 63.7 % of the total.

Finally, regarding social media website and application use, no significant differences were found between the general sample and the two sub-samples. In all cases,use was medium, varying between 57.1 % for nurses and 59.8 % for physicians.

### Discriminant factors of ECOPIH use

The results presented in the previous section highlight the existence of differences as regards technology use by the different healthcare professional profiles. In order to find out whether this tendency was repeated in relation to the level of ECOPIH use, a Chi-square analysis was performed. The results obtained show that, of the total sample of 166 individuals, only 47 % used ECOPIH. It was also found that, between the samples of physicians and nurses, there were significant differences in relation to the level of ECOPIH use (Chi-square = 6.458, sig. 0.008). Thus, 33.3 % of the nurses (19 individuals) compared to 54.1 % of the physicians (59 individuals) acknowledged using ECOPIH.

In order to identify the discriminant variables of ECOPIH use, both for the whole sample and the two sub-samples (physicians and nurses, respectively), a discriminant analysis was performed. Discriminant analysis is a multivariate statistical technique used when the dependent variable is categorical or nominal, and the independent variables are either metric or non-metric. The aim of this technique is to describe significant differences (if they exist) between g groups of objects (g > 1), on which p variables are observed (discriminant variables). To be more precise, the means of p classificatory variables are compared and described through the g groups defined by the dependent variable. As a result, this technique obtains the so-called discriminant function, which shows a linear combination of the independent variables that better discriminates between the groups defined *a priori*. Discrimination is performed by establishing the weightings of the theoretical value for each variable so as to maximise inter-group variance and minimise intra-group variance [48].

The small sample size and the relative newness of the platform prompted the use of an exploratory technique, which discriminant analysis is. The ultimate objective of discriminant analysis is to find the linear combination of independent variables that best distinguishes between several groups. The calculated discriminant function enables the probability of correctly classifying the individuals into one group or another to be increased [49]. Likewise, we also analysed the discriminant power of each factor, taking into account the role of the healthcare professional: physician vs. nurse.

Table 5 shows the goodness-of-fit and explanatory power statistics of the discriminant analyses performed for the whole sample and the two sub-samples, and the standardised coefficients of the calculated discriminant functions. The goodness of fit of the models was confirmed by the values and levels of significance reached by Box’s M test and the Chi-square statistic. Likewise, the eigenvalues of the discriminant functions were high, as were the canonical correlation values, thus indicating that the calculated function had high discriminant power.

**Table 5:** Standardised coefficients of the discriminant function

	Whole sample function	Physician sample function	Nurse sample function
Perceived usefulness for improving clinical practice quality	0.476***		0.523***
Perceived usefulness for reducing costs	0.547***	0.943***	-
Perceived ease of use	-	-	0.542***
Security and confidentiality	-	-	-
Mobile device use	-	-	-
Social media website and application use	0.237***	0.304***	-
Box’s M	46.720 (0.000)	26.227 (0.000)	25.74 (0.000)
Chi-square	104.974 (0.000)	53.835 (0.000)	55.394 (0.000)
Eigenvalue	0.908	0.662	0.648
Canonical correlation	0.690	0.631	0.626

***p = 0.000

Regarding the whole sample, we can see that the two dimensions making up perceived usefulness discriminate between the healthcare professionals that use the platform and those who do not. Specifically, perceived usefulness for reducing costs associated with clinical practice is the factor with the greatest discriminant power (β = 0.547). In descending order of importance, it is followed by perceived usefulness for improving clinical practice quality (β = 0.476), and lastly by habitual social media website and application use, which is also a factor that makes a greater contribution to the discriminant function, with a coefficient of β = 0.237. Thus, through both the high level of significance (99 %) as the value of β coefficients obtained by the variables *perceived usefulness for improving clinical practice quality and perceived usefulness for reducing cost*, it is possible to conclude that hypothesis H1 was confirmed. In addition, taking into account the result for the variable *social media website and application use*, it is possible to conclude that hypothesis H4.2 was confirmed. Finally, given the lack of significance of the results obtained, the rest of the hypotheses proposed in the theoretical model were rejected.

If we turn to the two sub-samples of healthcare professions, we can see that the usefulness stemming from ECOPIH use changes. Thus, for the physicians, perceived usefulness for reducing costs associated with clinical practice is the main factor when it comes to discriminating between using or not using the platform (β = 0.943). For the nurses, however, it is the perceived usefulness of the platform for improving clinical practice quality that ranks highest, and the one that has the biggest influence on their decision to use it (β = 0.523). Taking into account the values obtained for the variables *perceived usefulness for reducing cost* and *social media website and applications*, it is possible to conclude that hypotheses H1.2 and H4.2 were confirmed for the physicians.

Also in the case of the nurses, the factor that has greater discriminant power is perceived ease of use (β = 0.542). For the physicians, however, habitual social media website and application use is the second factor with discriminant power within the function (β = 0.304). Taking into account the values obtained for the variables *perceived usefulness for improving clinical practice quality and perceived ease of use*, and its high degree of significance, it is possible to conclude that hypotheses H1.2 and H4.2 were confirmed for the physicians.

To sum up, Table 6 shows the accepted and rejected hypotheses for each of the samples.

**Table 6:** Hypothesis confirmation

	Whole sample function	Physician sample function	Nurse sample function
*H1.1. The perception of improved clinical practice quality has an influence on the healthcare professional’s ECOPIH use.*	YES	NO	YES
*H1.2. The perception of reduced costs associated with clinical practice*	YES	YES	
*H2. The perceived ease of use of ECOPIH in clinical practice has an influence on the healthcare professional’s use of it.*	NO		YES
*H3. The perception of information security and confidentiality offered by ECOPIH use has an influence on the healthcare professional’s use of it.*	NO	NO	NO
*H4.1. The use of different mobile devices has an influence on ECOPIH use.*	NO	NO	NO
*H4.2. The use of different social media websites and applications has an influence on ECOPIH use.*	YES	YES	NO

## Discussion

The research revealed three main results. First, for the whole sample, the main discriminant factor that distinguished between ECOPIH users and non-users was the perception of reduced costs associated with clinical practice. It was followed by the healthcare professional’s perception of usefulness for improving clinical practice quality. Finally, habitual social media website and application use completes the configuration of the discriminant factors of ECOPIH use. These results suggest that the discriminant factors associated with organisational dynamics (reduced costs), clinical activity outcomes (improved quality) and with technology use are those that have the greatest influence on the healthcare professionals’ decision to use the platform. In this respect, it is important to note the importance of considering personal, organisational and outcome elements to foster ICT use in the field of healthcare.

Second, the results obtained from the discriminant analysis are clearly differentiated for the two sub-samples (physicians and nurses, respectively). For the physicians, the main discriminant factor was their perception of reduced costs brought about by using ECOPIH. For the nurses, however, the discrimination resulted from their perceptions of improved clinical practice quality and of the platform’s ease of use. As the TAM suggests, the statistical significance of these two discriminant factors refers to the importance of the perception of usefulness and ease of use when the use of a technology needs to be explained [32, 33]. On the other hand, this distinct discriminant orientation is probably the result of a different approach to using the platform, as a consequence of a care service provision that is also different. The physicians, with greater strategic and organisational responsibilities within the healthcare centres, see ECOPIH as a useful platform in the decision-making and cost-reduction process at a time of evident difficulties for the health system. In contrast, the nurses, whose approach to care is more pragmatic, rate the platform’s improved clinical practice quality and ease of use dimensions more highly.

Finally, habitual social media website and application use is of secondary importance in the configuration of the discriminant factors of ECOPIH use by the physicians. Although some physicians seem to be reluctant to move into social network applications, new models for staying abreast of and sharing medical knowledge with other physicians is needed due to the vast amount of medical knowledge required for patient care in PC fields [50]. It is, therefore, reasonable to assume that physicians are increasingly seeking alternatives for sharing information, and clinical VCoPs could provide an efficient and effective means of achieving this.

In most of the studies published to date, teleconsultation and data transfer is limited to Primary Care physicians and specialists without including other healthcare professionals, but our study provides new evidence because it finds that this perception of usefulness varies depending on the professional group analysed.

The research presented here has a number of limitations, particularly sample size, the constructs and indicators used, and the lack of a longer time series. Nevertheless, given the importance to the healthcare system of using Web 2.0 communication platforms like this one, and the availability of data for a group of professionals, it brings new evidence to the debate. Recommendations for future study therefore include increasing sample size, extending the analysis and incorporating new explanatory dimensions.

## Conclusions

In the context of communities of practice, two substantive conclusions can be drawn from this work. First of all, the perception of usefulness of Web 2.0 platform use for communication between primary and specialist healthcare professionals in the integration across primary and hospital care is determined by organisational, technological and social factors. Specifically, the position that professionals have within the healthcare structure and particularly the closer healthcare professionals’ activity is to patients and their professional experience of using social networks and ICTs are crucial to explaining the use of such platforms. Public policies promoting the use of ICTs in the field of medicine in general, and in communities of practice in particular, should therefore go beyond the purely technological dimension and consider other professional and social determinants, as well as those of an organisational and contextual nature.

Finally, a dynamic approach to the design of Web 2.0 communication platform use by PC professionals and specialists is needed, particularly when it targets a variety of end-users. Hence the importance of conducting studies prior to using such platforms, and attempting to identify which of the above-mentioned variables could exert an influence and how.

## Abbreviations

CoP: Community of practice
ECOPIH: Eina de Comunicació entre Primària i Hospitalària (Online Communication Tool between Primary and Hospital Care)
ICT: Information and communication technologies
PC: Primary care
TAM: Technology acceptance model
PCS: Primary Care Service
UOC: Open University of Catalonia
